# Temporal aspects of air pollutant measures in epidemiologic analysis: a simulation study

**DOI:** 10.1038/srep19691

**Published:** 2016-01-21

**Authors:** Laura F White, Jeffrey Yu, Michael Jerrett, Patricia Coogan

**Affiliations:** 1 Department of Biostatistics, Boston University School of Public Health, 801 Massachusetts Ave, 3^rd^ Floor, Boston, MA 02118 USA, (617)414-2833; 2 Slone Epidemiology Center, 1010 Commonwealth Ave, Boston MA 02115, USA; 3 Environmental Health Sciences Division (EHS), School of Public Health, University of California, Berkeley, 50 University Hall #7360, Berkeley, CA 94720-7360, USA

## Abstract

Numerous observational studies have assessed the association between ambient air pollution and chronic disease incidence, but there is no uniform approach to create an exposure metric that captures the variability in air pollution through time and determines the most relevant exposure window. The purpose of the present study was to assess ways of modeling exposure to air pollution in relation to incident hypertension. We simulated data on incident hypertension to assess the performance of six air pollution exposure metrics, using characteristics from the Black Women’s Health Study. Each metric made different assumptions about how to incorporate time trends in pollutant data, and the most relevant window of exposure. We use observed values for particulate matter ≤2.5 microns (PM_2.5_) for this cohort to create the six exposure metrics and fit Cox proportional hazards models to the simulated data using the six metrics. The optimal exposure metric depends on the underlying association between PM_2.5_ and disease, which is unknown. Metrics that incorporate exposure information from multiple years tend to be more robust and suffer from less bias. This study provides insight into factors that influence the metric used to quantifying exposure to PM_2.5_ and suggests the need for careful sensitivity analyses.

Numerous observational studies have assessed the association of ambient air pollution with mortality and chronic disease incidence. Associations between ambient levels of particulate matter of ≤2.5 microns in aerodynamic diameter (PM_2.5_) and all-cause or cause-specific cardiovascular mortality have been observed in the American Cancer Society Cohort^1^, the Harvard Six Cities Study^2^, the Women’s Health Initiative^3^, and many other studies^4^. Hazard ratios tend to be at the lower limit of detection for observational studies (e.g., 1.04 and 1.14 per 10 μg/m^3^ increase in PM_2.5_ for all-cause mortality in the American Cancer Society study^1^ and the Harvard Six Cities Study^5^, respectively); the highest hazard ratios (HRs) are near two (e.g., 2.02 per 10 μg/m^3^ increase in PM_2.5_ for ischemic heart disease in the Nurses’ Health Study)^6^. Associations have also been found with chronic disease incidence, including atherosclerosis^7^,^8^, diabetes^9^,^10^,^11^, and hypertension^12^, with hazard ratios in the range of 1.1–1.3 per increment change in exposure, while other studies have found no association with chronic disease^13^,^14^.

One reason for varying results may be differences in the methods used to assess ambient pollution levels, which include assignment based on nearest pollution monitor^1^, geostatistical interpolation^15^, land use regression models^16^,^17^, dispersion models^18^, and satellite-based estimates^10^. The different estimation methods, their applicability to short and long-term health studies, and their limitations, have been discussed previously^17^,^19^,^20^,^21^,^22^,^23^.The purpose of the present paper is to assess another possible reason for inconsistent study results: different ways of translating the pollutant estimates into exposure metrics appropriate for Cox proportional hazards modeling. This translation involves choices about whether or how to incorporate time trends in the pollutant data and the definition of the relevant exposure period. The first choice addresses the question of how to include the maximum follow-up time for a cohort when ambient measures are available only for a small portion of follow-up. The second choice addresses the question of how far back is exposure relevant and when does exposure cease being relevant.

In time-series studies of short term air pollution exposures and acute outcomes, like stroke or emergency room admissions, the relevant exposure period is assumed to be within days of the event, and the impact of exposure on different days can be easily assessed. In contrast, the exposure period that is relevant to the onset of a chronic disease is not known. One can only assume that the relevant exposure period is probably years, not months, and that exposure ceases to be relevant at some point because subclinical disease has already occurred. The assumptions that define the relevant exposure period are embodied in the way that the measures of ambient pollutant levels are incorporated into the regression model as exposure metrics.

Researchers have taken different approaches. In some follow-up studies, including the American Cancer Society study of PM_2.5_ and mortality^1^, and Canadian and German studies of PM_2.5_ and diabetes incidence^9^,^10^, the main exposure metric was the long-term average concentration of PM_2.5_ at the subject’s baseline location, with no temporal variation over follow-up. Other longitudinal studies have averaged monthly or daily pollutant measures from different time periods. For example, in the Nurse’s Health Study of PM_2.5_ and diabetes incidence, the main exposure metric was monthly PM_2.5_ values averaged over the 12 months before diabetes diagnosis or end of follow-up^14^. In the Danish Diet, Cancer, and Health cohort study of nitrogen oxides and hypertension incidence, the exposure was modeled as 1- and 5-year time-weighted averages before diagnosis or end of follow-up^13^.

The purpose of the present paper is to evaluate six exposure metrics that represent different ways of accounting for time-trends in pollutant data and various assumptions about relevant exposure periods. We assessed the metrics using two sets of simulated data, each of which makes a different assumption about the true association of air pollution exposure and disease. We then compared the magnitude and precision of the hazard ratios derived from models using the six different metrics. We used ambient PM_2.5_ data generated for an ongoing longitudinal study of PM_2.5_ and the incidence of diabetes and hypertension, and simulated data on disease incidence.

## Methods

To construct the six exposure metrics, we used ambient PM_2.5_ data generated for a study of air pollution and hypertension and diabetes incidence in the Black Women’s Health Study (BWHS). The BWHS is a follow-up study initiated in 1995 when 59,000 women in the United States were recruited via a mailed health questionnaire. Every two years, the cohort members complete questionnaires to report incident disease and update data on risk factors and residential addresses. PM_2.5_ estimates have been linked to participant residential addresses current at each 2-year questionnaire cycle from 1999-2008; we back calculated to 1995 for the simulations, as described below. As women move, their pollution exposure is updated. We use information on the incidence of hypertension in BWHS and its relation to age, body mass index (BMI) and neighborhood socioeconomic status (SES, estimated from census data) to inform the parameters used in the simulation. The study protocol was approved by the Institutional Review Board of Boston University School of Medicine and the methods were carried out in accordance with approved guidelines. Participants indicate informed consent by completing and returning the questionnaires.

### Estimation of ambient PM_2.5_

We used a hybrid modeling approach to estimate monthly PM_2.5_ levels at BWHS residential addresses in 1999,described in detail elsewhere^16^. In brief, we used a two-stage modeling strategy that incorporated a land use regression (LUR) approach and a Bayesian maximum entropy (BME) approach. We developed the models with PM_2.5_ measurements from the U.S. Environmental Protection Agency’s Air Quality System national network of 1464 monitoring locations. The final data set of 104,172 monthly PM_2.5_ measures, from January 1999 through December 2008, was partitioned into a training set for modeling-fitting purposes (90%) and a cross-validation dataset (10%) for model validation. We first used LUR to construct a deterministic-like model attributing various measures of traffic, land use, and population to the variability in PM_2.5_ measures, using a machine learning approach to select the final model. We then applied BME methods to the spatio-temporal residuals from the LUR model using the expected distribution of the predicted values as prior information. The “residual surface” described the spatiotemporal variability of the pollution estimates that could not be described by the LUR model. Validation of the final LUR-BME model in the reserved cross-validation dataset showed strong agreement between observed and predicted PM_2.5_ levels with no evidence of substantial bias; the *R*^*2*^ was 0.79.

### Data Simulation

We simulated data on the incidence of hypertension, but results are applicable to any chronic illness with a long latency period and for which, relative to air pollution, there is a long induction period. To simulate the data, two assumptions must be made: (1) the baseline characteristics that predict hypertension incidence in the population, and (2) the true nature of the relationship between PM_2.5_ and hypertension incidence. For assumption 1, we have assumed that the incidence of hypertension is related to age, BMI and SES at the start of follow-up (1999), as is the case in BWHS^24^,^25^. We assumed the event times follow a Gompertz distribution with scale parameter of 0.0002 and shape parameter of 0.49 to most accurately reflect the observed time to event.

**Figure 1:**
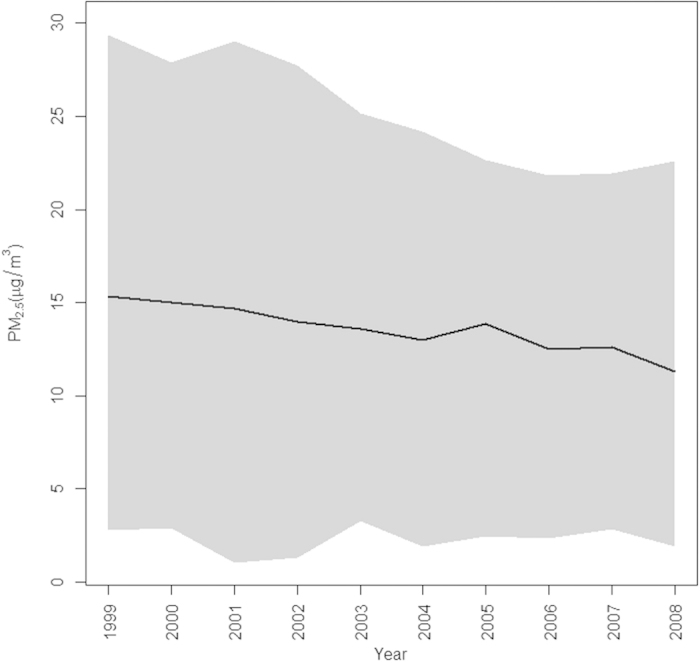
Annual PM_2.5_ measures for the cohort from 1999 to 2008. The black line is the average PM_2.5_ value and the shaded area shows the range of values observed for the entire cohort.

For assumption 2, we assumed that the incidence of hypertension is positively related to PM_2.5_, and that hypertension incidence over time reflects change in PM_2.5_ levels through time. In BWHS data, PM_2.5_ at most locations decreases in an approximately linear fashion through time (Fig. 1). We simulated survival times assuming two different relationships between PM_2.5_ and the time to event. In both scenarios we simulate hypertension incidence assuming a linear change in PM_2.5_ through time, using the method described by Austin (see appendix)^26^. Thus each woman in the simulation has an individually estimated linear change in PM_2.5_, and her hazard of developing hypertension at any time is a function of the hazard distribution, her baseline BMI, SES, age, and the linearly changing PM_2.5_ at her address. In the first scenario we fit a linear regression to each woman’s PM_2.5_ values through time and associated the best linear fit of PM_2.5_ with outcomes one year later. Therefore, an outcome in year t is best predicted by the value of PM_2.5_ one year prior. In the second scenario, we assumed that the cumulative exposure to PM_2.5_, from start of follow up to diagnosis or end of follow-up, has the most influence on disease occurrence. In this case we fit a linear regression model to the time-varying cumulative average of PM_2.5_ for each woman and used this linear assumption of exposure to simulate survival times for each woman.

In both scenarios, those whose simulated survival time preceded the end of follow-up (2008) were randomly assigned an indicator of whether the individual was a case or censored using a uniform distribution with a probability of being a case set to 0.6, reflecting the proportion observed year to year in BWHS among women whose time to follow-up was less than the study period. In other words, those with simulated follow-up less than 10 years have a 60% chance of being a case and a 40% chance of being censored. This rate is fairly consistent through time in BWHS data.

For each simulated dataset, we postulated three hazard ratios (HR) describing the association of PM_2.5_ with hypertension: 1.0, 1.2, or 1.5, for a total of 6 simulation scenarios (three HRs and two sets of simulated data). We simulated 1000 datasets for each scenario.

### Exposure metrics

We explored six exposure metrics. Table 1 illustrates how the metrics would be calculated for disease diagnosis occurring in 2005. The columns show the weights assigned to pollutant measures each year; the weights reflect when exposure is considered relevant, and which years are considered critical exposure periods. The first two metrics do not vary through time and represent two commonly used approaches: the baseline value in 1999 (baseline mean) and the mean of PM_2.5_ over follow-up 1999-2008 (overall mean). The weights assigned indicate that, for the baseline mean, only the average annual pollutant level in 1999 is taken into account, and for the overall mean, all annual averages have equal weight. The next approach (cumulative average), is the average of annual average pollutant values that occurred prior to the year of diagnosis, weighted equally. The weighted average incorporates values in all years prior to diagnosis but weights values from years closest to diagnosis more heavily. Next, the “1-year prior” and “3-year prior” metrics reflect a common approach in air pollution epidemiology wherein the exposure metrics are unweighted averages from various periods preceding diagnosis. All but the first two metrics and the 3-year prior metric include pollutant estimates up to the year prior to diagnosis (2004) – thus assuming that exposures encountered in the year of diagnosis (2005 in our example) are not relevant. The 3 year prior metric assumes that pollutant levels encountered one and two years before diagnosis are not relevant. Clearly there could be many combinations of averaging periods and weightings– we have chosen these to represent a wide range approaches.

### Assessment of Exposure Metrics

We compared the HRs estimated using the six exposure metrics with the postulated HRs by fitting a Cox proportional hazards model to each of the simulated datasets, adjusted for baseline age, BMI and neighborhood SES. The exposure metrics were treated as time varying covariates in the model, except for the first two metrics, which did not vary with time. We report the estimates of the HRs from the simulated data and the width of the confidence intervals are included as a measure of precision. We also show results for the “true” exposure metric from both simulation scenarios, which is the one year lagged linear estimate of exposure for the first simulation scenario and the linear estimate of the cumulative average for the second scenario (labeled “True Exp” in the results). This is reasonably close to the “true” exposure assumed in the data. All simulations and analyses were performed using R 2.15.2 (r-project.org). All code is available from the first author upon request.

## Results

The baseline values of PM_2.5_ ranged from 2.74 *μg/m*^*3*^ to 29.38 *μg/m*^*3*^ with a median value of 15.45 *μg/m*^*3*^. Over time, most individual values of PM_2.5_ decreased from their baseline value with an estimated median linear rate of change per year of −0.38 *μg/m*^*3*^ (range: −1.54 *μg/m*^*3*^ to 0.35 *μg/m*^*3*^) (Fig. 1).

**Figure 2:**
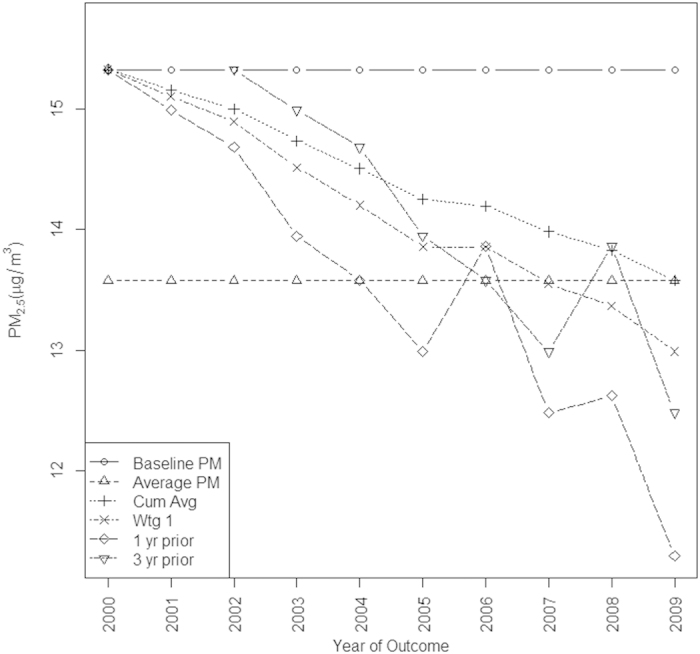
Exposure metrics plotted by the year of assessment, averaged over the entire cohort.

**Figure 3:**
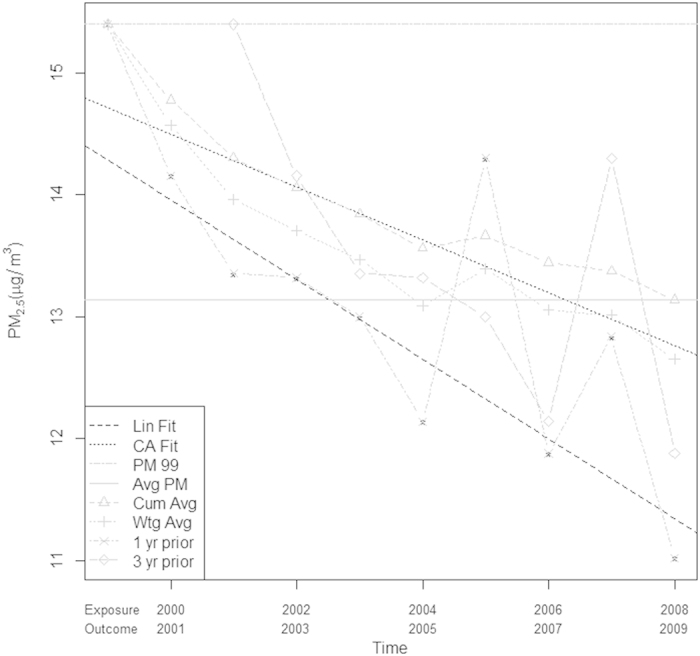
Simulation schemes with exposure metrics to be evaluated for one randomly chosen woman in the cohort. Lin Fit and CA fit show the linear assumptions used in each of the simulation scenarios. The gray lines show the values of each of the six exposure metrics through time.

**Figure 4:**
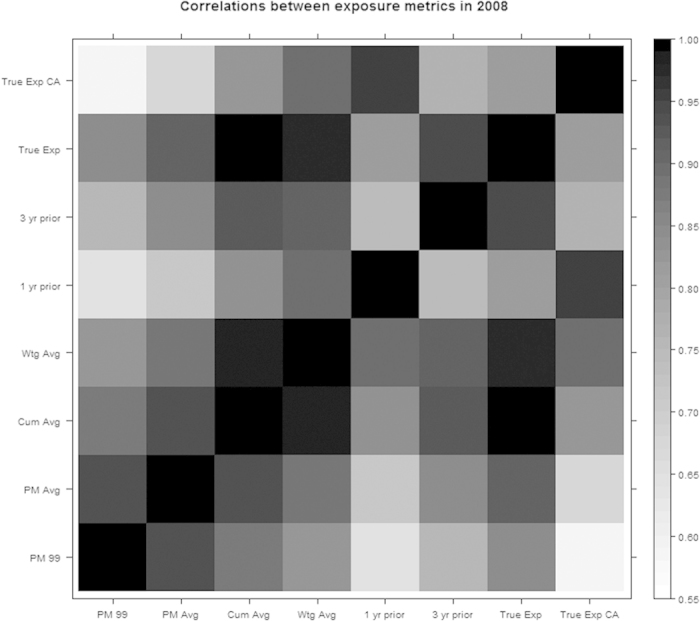
Spearman correlations between exposure metrics in 2008. The legend on the right illustrates the scale used to show the strength of the correlation, which darker colors indicating greater correlation.

Figure 2 illustrates the average values of PM_2.5_ that the exposure metrics assign to the simulated observations through time. The baseline value and the overall average values do not change with time, while the one and three year prior values show the greatest change through time, since they track the raw PM_2.5_ values. Figure 3 shows the relationship between these exposure metrics and the assumptions of the two simulation scenarios for one randomly chosen woman. We note that in general the time varying average metrics overestimate the true exposure for the first simulation scenario. The exposure metrics were highly correlated with one another. Spearman correlation coefficients for the metrics in 2008 are shown in Fig. 4. The r for baseline PM_2.5_ and overall average of PM_2.5_ was 0.94. These two time-invariant metrics were reasonably highly correlated with the cumulative average and the weighted average (r = 0.82–0.93); correlations were slightly less for the 3- year prior metric. The cumulative and weighted averages were all highly correlated with one another (r = 0.99), but slightly less correlated with the 3 year prior measure (r = 0.92, 0.91).

**Figure 5:**
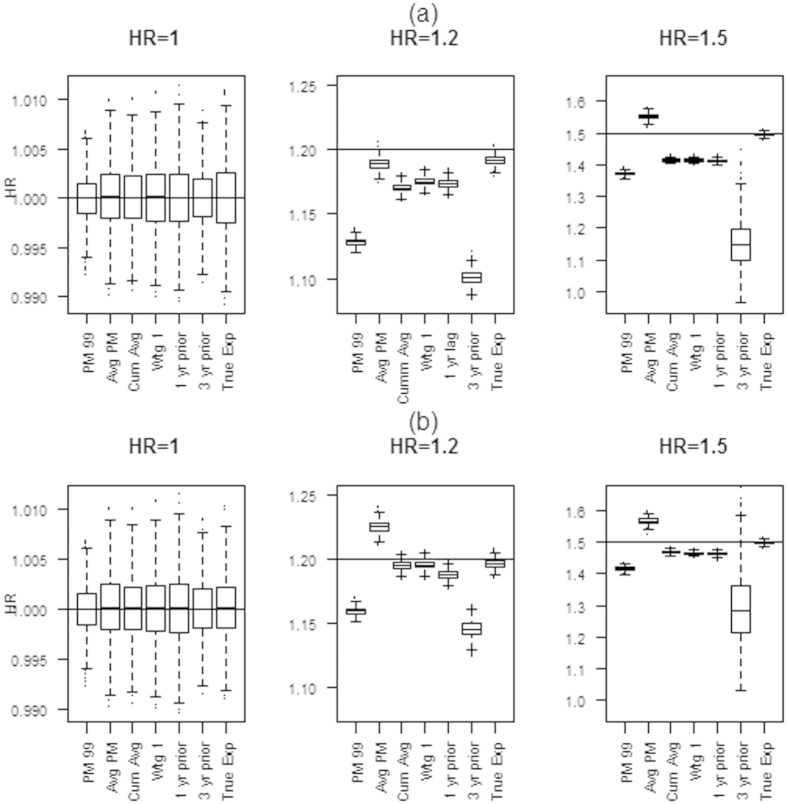
Hazard Ratios from the simulated data with time varying PM_2.5_ exposure following linear pattern. (**a**) with a one year lag between PM_2.5_ exposure and outcome, and (**b**) based on the cumulative average. Boxplots shows median, IQR and range of the estimated HRs from each of the 1000 simulations for each exposure metric.

Figure 5 shows results from fitting Cox models to the simulated data. Figure 5(a) shows the hazard ratios estimated under simulation scenario 1 (assuming a linear change in PM_2.5_ that follows the one year lagged PM_2.5_ for each participant). Figure 5(b) shows the hazard ratios under simulation scenario 2 (assuming a linear change in PM_2.5_ that follows the cumulative average). The plots show the median, the 1st and 3^rd^ quartiles (limits of box), and range (ends of dotted lines), of the HRs from the 1000 simulations. The HRs generated under each of the 2 simulation scenarios are similar. Under both scenarios, when the postulated true HR = 1.0, all metrics yielded a HR of 1.0, as expected. When the HR exceeds 1.0, choice of metric becomes more consequential. The first metric, the baseline PM_2.5_ (PM99), consistently underestimated the HR for both simulation scenarios. The other time-invariant metric, the average PM_2.5_ (Avg PM), performed better, but tended to overestimate the true HR for all scenarios, with one exception (when the true HR was 1.2 and the data was simulated to follow the one year lagged PM_2.5_). The cumulative average (Cum Avg), weighted average (Wgt) and one year prior metric performed reasonably well in all scenarios. Whereas the results are similar for both simulation scenarios, we observed slightly more bias in the first simulation scenario, (true exposure is lagged by one year). When the true exposure follows the cumulative average, the cumulative and weighted averages performed much better. This is not surprising after close inspection of Fig. 3. We note that generally the cumulative average decreases at a slower rate than the raw data, thus the averaged exposure metrics will tend to be higher than the true exposure in the first simulation scenario. The three year prior metric, like the baseline PM_2.5_, systematically underestimated the true HR. The true exposure metric provides a useful benchmark to evaluate the performance of the exposure metrics and closely follows the assumed HR, providing validity to the simulations.

As regards precision of the estimates, the widths of the confidence intervals were small for all metrics (within three decimal places of 0.010), except for the three year prior metric, where the width was slightly greater (supplementary Figure 1).

## Discussion

We have presented results from a simulation study to assess the sensitivity of hazard ratios calculated with Cox proportional hazards models to the choice of air pollution exposure metric. We evaluated the scenario where each participant can move over the course of follow-up and the outcome is a chronic illness. In our simulations, we used PM_2.5_ as the pollutant and assumed that its relation with the risk of illness changed linearly through time following either the trend of the raw data, lagged by one year (simulation scenario 1) or the trend of the cumulative average of the raw data (simulation scenario 2). Our results indicate that using averages of PM_2.5_ values that occur prior to the time when the hazard is being estimated provided the most robust estimator of exposure. When the baseline value of PM_2.5_ or the three year prior value of PM_2.5_ was used, the models consistently underestimated the HR. The time invariant average PM_2.5_ (which includes pollutant levels that occurred after disease diagnosis) performed reasonably well in all scenarios, though always with some bias.

We assumed that PM_2.5_ is a linearly time varying risk factor for chronic illness. While the true underlying relationship between PM_2.5_ and any outcome is unknown, we believe our assumptions are reasonable and provide insight on the factors to consider when choosing an exposure metric. We have reported on two scenarios that postulate a relationship between PM_2.5_ and the outcome. It is possible to hypothesize more complex simulation scenarios and other relationships between the exposure and outcome, as well as the role of confounding factors. However, they would be equally speculative and it is not clear how to parameterize them^27^. Additionally, our goal in this project was to inform the choice of exposure metrics in follow-up studies of air pollution and chronic disease incidence. In the absence of an understanding of the true relationship between the exposure and outcome, we are left to choose a metric that appears most robust. Our results advocate for sensitivity analyses and careful discussion of the potential relationship between exposure and outcome under various assumptions of the true relationship between the exposure and outcome.

One implication of our findings is that metrics that incorporate exposure information from multiple years are superior to metrics using information from a single year. While multi-year ambient measures can be combined into a variety of exposure metrics, they tend to be highly correlated, and to give similar effect estimates. This has been found in other studies. For example, in a sensitivity analysis of the Six Cities Study^28^, the authors concluded that “Attempts to identify critical exposure time windows were limited by the lack of marked interindividual variation in temporal exposure patterns throughout the study period”^14^. Other studies reported similar HRs regardless of averaging period of the air pollution data, including null studies^13^,^14^ and studies where a positive association was found between air pollution and the outcome^12^,^29^. One might argue that the 1 year lagged exposure metric performs equally well, however it tends to have higher variation and when the true exposure is based on the cumulative average, it begins to show more bias. However, this metric does exhibit reasonably high correlation with the averaged metrics, and therefore might be an acceptable substitute when limited data is available.

## Conclusions

Our simulation study illustrates the potential impact of the choice of exposure metric on the estimated hazard ratio. In the scenarios shown, averaged values of exposure tend to perform better than a single observation from one year.

## Additional Information

**How to cite this article**: White, L. F. *et al*. Temporal aspects of air pollutant measures in epidemiologic analysis: a simulation study. *Sci. Rep.* **6**, 19691; doi: 10.1038/srep19691 (2016).

## Supplementary Material

Supplementary Information

## Figures and Tables

**Table 1:** Exposure Metrics Used in the Study.

Year	1999	2000	2001	2002	2003	2004	2005	2006	2007	2008
Metric	Weights Assigned									
Baseline PM	1	0	0	0	0	0	0	0	0	0
Overall average, 1999–2008	0.1	0.1	0.1	0.1	0.1	0.1	0.1	0.1	0.1	0.1
Cumulative average	0.167	0.167	0.167	0.167	0.167	0.167	0	0	0	0
Weighted	0.05	0.10	0.14	0.19	0.24	0.29	0	0	0	0
Previous year	0	0	0	0	0	1	0	0	0	0
Three years prior	0	0	0	1	0	0	0	0	0	0

The weights show how pollutant measures each year were weighted in calculating the exposure metric for disease diagnosis occurring in 2005. For instance, the cumulative average metric would equally weight all observations in the years 1999-2004, while the weighted metric places emphasis on observations closer to 2005.
