# Supplementary material for: Temporal aspects of air pollutant measures in epidemiologic analysis: a simulation study
Source: Sci Rep. 2016 Jan 21;6:19691. doi: 10.1038/srep19691 (PMC4726372; doi:10.1038/srep19691)
Supplement: Supplementary Information [file srep19691-s1.pdf]

Supplementary Information

Temporal aspects of air pollutant measures in epidemiologic analysis: a simulation study

Running title: Temporal aspects of air pollutant measures

Laura F White, PhD[1*], Jeffrey Yu, MPH[2], Michael Jerrett, PhD[3], Patricia Coogan, ScD[2]

Here we explain the simulation method of Austin[1] in more detail. This method allows one to simulation survival data with a time varying covariate and builds on previous work by Bender et al.[2] Bender shows that survival time can be simulated using the function $T = H_0^{-1}[-\log(u)\exp(-\beta' x)]$, where $u \sim U(0,1)$, the standard uniform distribution and x are the covariates in the model with β' being the assumed log of the hazard ratio. Thus one need only generate a standard uniform number, u, and then use the formula to get a survival time given a covariate value x, and assumed value for the log of the hazard ratio, β. In this case $H_0()$ is the cumulative baseline hazard function. Bender has shown that the Weibull, Exponential and Gompertz parametric forms for the survival times assume proportional hazards, similar to the popular semi parametric Cox model. The exponential distribution requires only a single parameter and is simple to implement, but might be unrealistic in many settings. The Weibull and Gompertz distributions use two parameters and, while more complex, offer greater flexibility to more accurately portray a distribution more realistically.

Austin describes how to simulate survival times for all three of these parametric distributions for the following types of covariates:

1. dichotomous time-varying covariate with at most one change (eg. moving from treated to untreated), and
2. continuous time-varying covariate.

Our simulation assumes the latter case. Austin typically assumes that the exposure is increasing with time, as would occur with an individual being exposed to more and more

drug with time on a drug trial. In our setting, pollution exposure has typically decreased through time. This fact, coupled with the complex distribution of the survival times led us to assume a Gompertz distribution of the survival times. The more simple exponential distribution was inadequate for our scenario and the Weibull did not perform well with a decreasing exposure.

If we assume that the exposure to pollution is changing linearly with time, then we can describe our exposure by the simple relationship, $x(t) = b_0 + kt$, where k describes the linear change with time t and b_0 is the value of the pollutant at baseline. We slightly modify the work of Austin to incorporate the intercept and obtain the following formula for simulating survival times:

$$T = \frac{1}{\beta_t k + \alpha} log \left[1 + \frac{(\beta_t k + \alpha)(-\log(u))}{\lambda \exp(\beta' x + \beta_t b_0)} \right],$$

where $\beta' x$ are the other covariates included in the analysis. In our case, these are age, SES, and BMI and β' is estimated using a Cox mode with the original data. The parameters α and λ characterize the Gompertz distribution and were estimated from the observed survival times.

1. Austin, P. Generating survival times to simulate Cox proportional hazards models with time-varying covariates. *Stat. Med.* **31,** 3946–3958 (2012).

2. Bender, R., Augustin, T. & Blettner, M. Generating survival times to simulate Cox proportional hazards models. *Stat. Med.* **24,** 1713–1723 (2005).

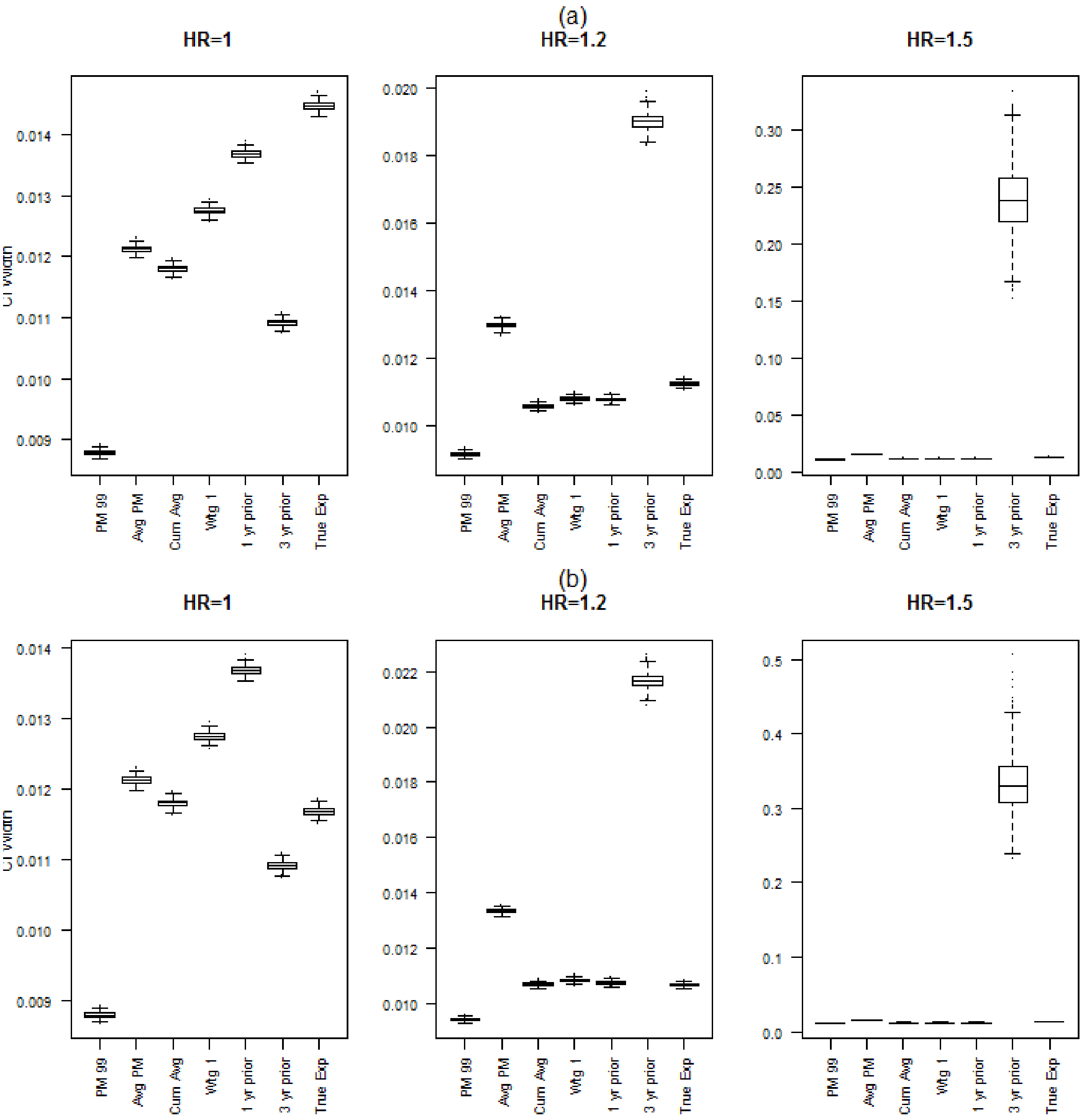

Figure S1. The width of the confidence interval from the simulated data with time varying $PM_{2.5}$ exposure following linear pattern (a) with a one year lag between $PM_{2.5}$ exposure and outcome, and (b) based on the cumulative average. Boxplots shows median, IQR and range of the confidence interval width from each of the 1000 simulations for each exposure metric.
